# Supplementary material for: Binding partners regulate unfolding of myosin VI to activate the molecular motor
Source: Biochem J. 2022 Jul 4;479(13):1409–28. doi: 10.1042/BCJ20220025 (PMC9342898; doi:10.1042/BCJ20220025)
Supplement: Supplementary Material [file BCJ-479-1409-s1.pdf]

Supplementary Table 1. Recombinant DNA.

Construct (Residue numbers)	Source
Xenopus pFastBac1 Calmodulin (1-end)	J. Sellers (NIH)
Human pET151 NDP52 (1-end)	Ref Fili et al 2017
Human pET151 EGFP-$MVI_{TAIL(NI)}$-RFP (814-1253)	Ref Fili et al 2017
Human pET151 EGFP-$MVI_{TAIL(LI)}$-RFP (814-1284)	Synthetic Gene – This study
Human pET151 MVI_{TAIL} 814-1253	Ref Fili et al 2017
Human pET151 MVI_{TAIL} 814-1060(NI)	Ref Fili et al 2017
Human pET151 MVI_{TAIL} 814-1091(LI)	Synthetic Gene – This study
Human pET28 CBD 1060-1253	Ref Fili et al 2017
Human pFastbacHTB NI MVI (1-1253)	Ref Fili et al 2017
Human pFastbacHTB MVI (1-814)	Ref Fili et al 2017
Human pFastbacHTB MVI (1-1060)	Ref Fili et al 2017
Xenopus pET28 Cys Calmodulin	Ref Fili et al 2017
Human pET151 Dab2 (649-770)	Ref Fili et al 2020
Human pEGFP-C3 CBD 1060-1253	F. Buss (CIMR)
Human pLV-Tet0-Halo-MVI RRL/AAA	This study
Human pLV-Tet0-Halo-MVI WWY/WLY	This study
Human pEGFP-C3 NI MVI	Ref Fili et al 2017
Human pEGFP-C3 NI MVI RRL/AAA	Ref Fili et al 2020
Human pEGFP-C3 NI MVI WWY/WLY	Ref Fili et al 2020
Human pcDNA3.1 Halo-MVI	Synthetic Gene – This study

Supplementary Table 2. PCR Primers.

Sequence	Use
CTTGCAGAGAAGAATTTCATGCGGCAGCAAAAGTGTATCATGC	RRL/AAA For
GATTTCCAAGCATGATACACTTTTGCTGCCGCATGAAATTCTTC	RRL/AAA Rev
CCCTCAGAGTAAGAAAAAAAGGCTGGTTGTATGCCCATTTTGA	WWY/WLY For
GGTCCATCAAAATGGGCATACAACCAGCCTTTTTTCTTACTCT	WWY/WLY Rev

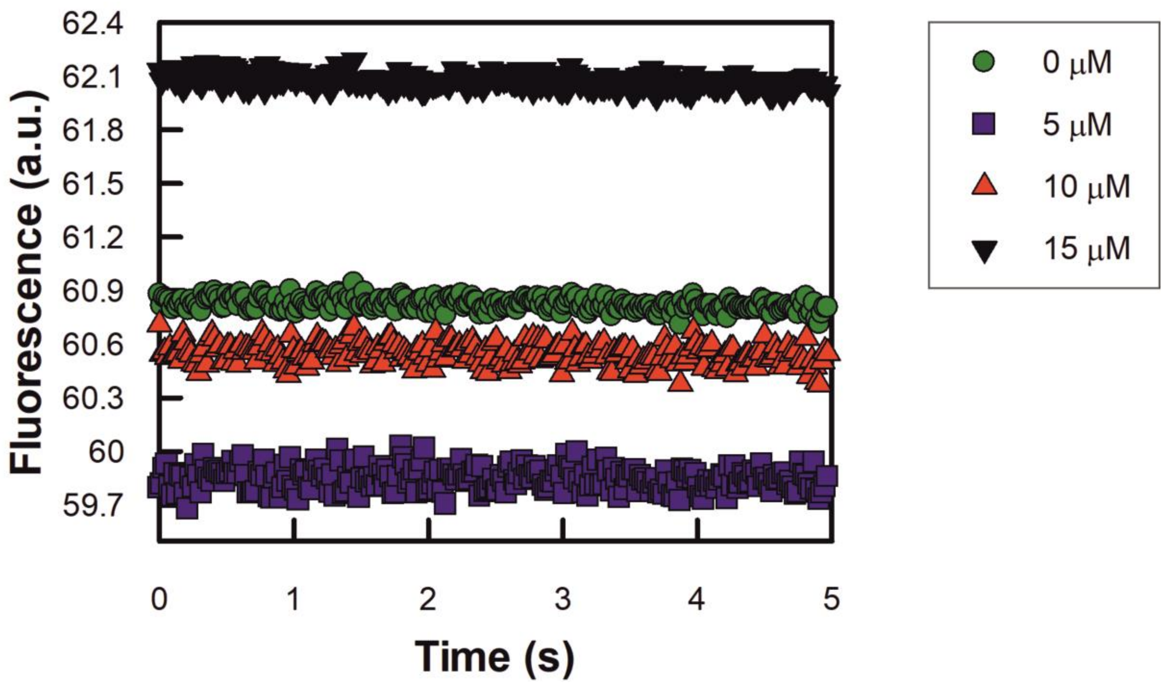

Supplementary Figure 1. Representative stopped-flow fluorescence traces following rapid mixing of 1 μM Cy3B-calmodulin and unlabelled NDP52 at the stated concentrations. Experiments were performed as described in the methods.

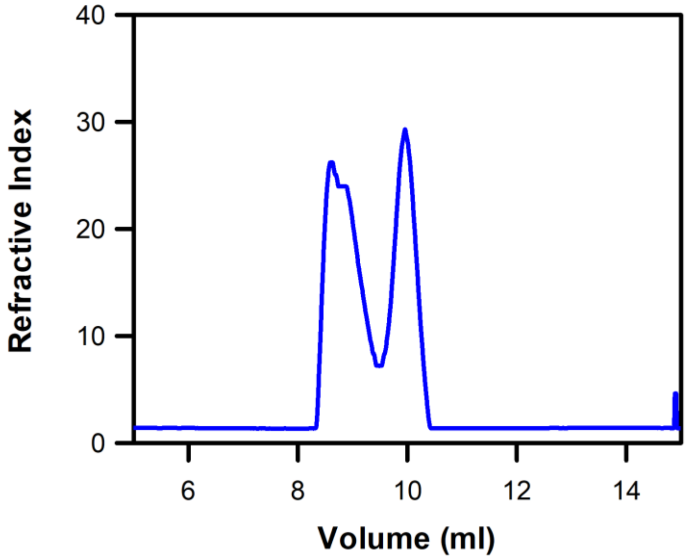

Supplementary Figure 2. Representative Size exclusion chromatography trace for 2mg/ml NDP52 where a large peak outside the column limits is possible to observe.

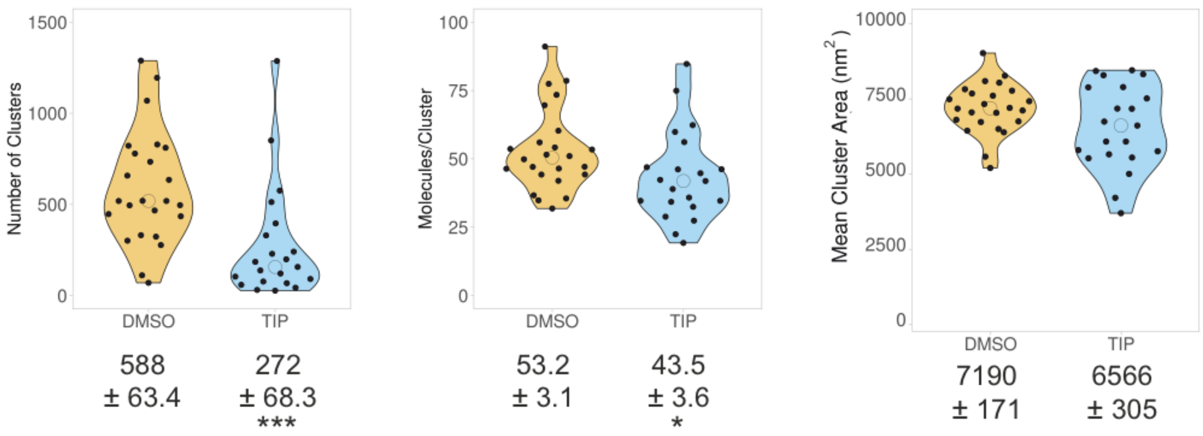

Supplementary Figure 3. Cluster analysis of MVI in the presence and absence of TIP, as described in Figure 5. Individual data points correspond to the average number of molecules per cluster in the selected ROI, in an individual cell. The values represent the mean from all the ROIs for each protein (n = >20). (*p <0.05 ***p <0.001 by two-tailed t-test).

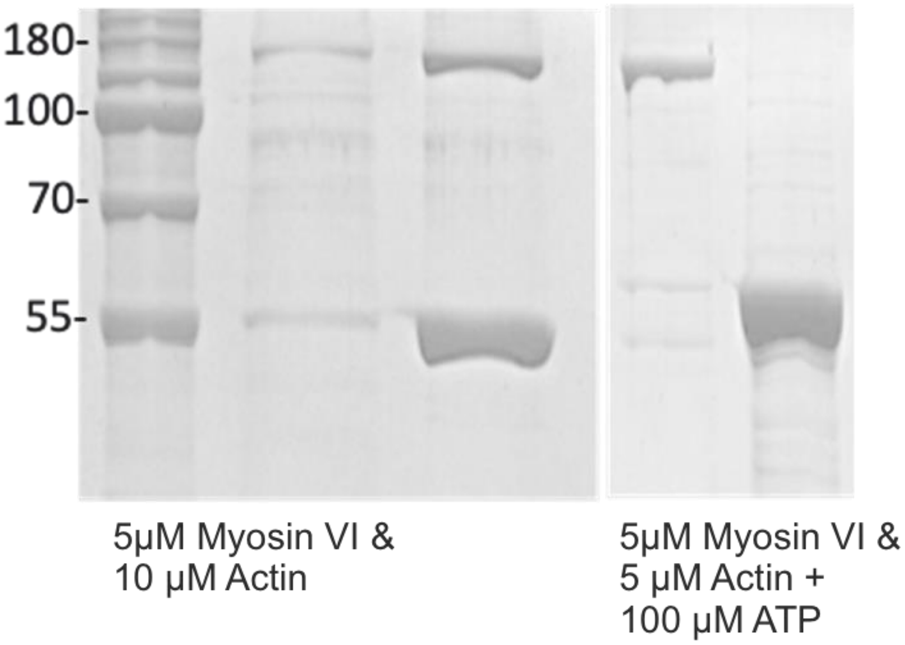

Supplementary Figure 4. Actin pull-down of (*left*) 5 µM myosin VI wild type and 10 µM Actin preparation. (*right*) 5 µM myosin VI wild type and 5 µM Actin preparation in the presence of 100 µM ATP. P and S represent pellet and supernatant, respectively.
